# Measurement of relaxation times in extensional flow of weakly viscoelastic polymer solutions

**DOI:** 10.1007/s00397-016-0980-1

**Published:** 2016-11-19

**Authors:** Patrícia C. Sousa, Emilio J. Vega, Renato G. Sousa, José M. Montanero, Manuel A. Alves

**Affiliations:** 1 Departamento de Engenharia Química, CEFT, Faculdade de Engenharia da Universidade do Porto, Rua Dr. Roberto Frias, 4200-465 Porto, Portugal; 2 Departamento de Ingeniería Mecánica, Energética y de los Materiales and Instituto de Computación Científica Avanzada (ICCAEx), Universidad de Extremadura, E-06006 Badajoz, Spain

**Keywords:** Extensional relaxation time, Viscoelastic fluid, Uniaxial extension, Elongational flow

## Abstract

The characterization of the extensional rheology of polymeric solutions is important in several applications and industrial processes. Filament stretching and capillary breakup rheometers have been developed to characterize the extensional properties of polymeric solutions, mostly for high-viscosity fluids. However, for low concentration polymer solutions, the measurements are difficult using available devices, in terms of the minimum viscosity and relaxation times that can be measured accurately. In addition, when the slow retraction method is used, solvent evaporation can affect the measurements for volatile solvents. In this work, a new setup was tested for filament breakup experiments using the slow retraction method, high-speed imaging techniques, and an immiscible oil bath to reduce solvent evaporation and facilitate particle tracking in the thinning filament. Extensional relaxation times above around 100 μs were measured with the device for dilute and semi-dilute polymer solutions. Particle tracking velocimetry was also used to measure the velocity in the filament and the corresponding elongation rate, and to compare with the values obtained from the measured exponential decay of the filament diameter.

## Introduction

The capillary thinning and breakup of Newtonian and viscoelastic liquid filaments are considerably different (Anna and McKinley 2001; Oliveira and McKinley 2005). The filament thinning is triggered a capillary instability (Anna and McKinley 2001) and the subsequent evolution of the filament thread for Newtonian fluids is the result of the competition between the driving surface tension, viscosity, and inertial effects, with the filament diameter decreasing linearly with time in the last stage of the breakup process (Entov and Hinch 1997; Vega et al. 2014).

Adding even a small amount of a high molecular weight polymer to the fluid has a significant effect on the filament thinning and breakup (Goldin et al. 1969). Although the process is also initiated by a capillary instability, the presence of the polymer macromolecules generates a quasi-cylindrical filament, which takes more time to thin and pinch off (Middleman 1965; Goldin et al. 1969). During the thinning of viscoelastic liquid filaments, elastic and capillary forces balance each other, while inertial, viscous, and gravitational effects are often negligible. In this elasto-capillary regime, the filament diameter decreases exponentially with time (Entov and Hinch 1997), and tensile stresses grow exponentially because polymeric chains are elongated at a constant extensional rate, $\dot {\varepsilon }= 2/(3{\lambda })$, where *λ* is the liquid extensional relaxation time (Bazilevsky et al. 1990). The formation of structures can occur during the thinning of the viscoelastic filament, such as the beads-on-a-string (BOAS) phenomenon (Bhat et al. 2010).

The measurement of the extensional properties of complex fluids is of great relevance for industrial processes such as inkjet printing, fiber spinning, spraying, and atomization. Several studies have used filament and capillary breakup rheometers (Bazilevsky et al. 1981; Bazilevsky et al. 1990; Matta and Tytus 1990; Stelter et al. 2000; Nelson et al. 2011; Dinic et al. 2015) to investigate different aspects of the problem, such as the velocity profile during the thinning of viscoelastic filaments (Gier and Wagner 2012); the effect of the molecular weight and concentration of the polymer on the filament thinning and breakup (Clasen et al. 2006; Tirtaatmadja et al. 2006; Arnolds et al. 2010); the BOAS instability (Oliveira and McKinley 2005; Bhat et al. 2010); the effect of the mass transfer resulting from water absorption in hygroscopic fluids leading to a change of the extensional viscosity (McKinley and Tripathi 2000); the measurement of both the relaxation time and extensional viscosity of viscoelastic fluids (McKinley and Tripathi 2000; Stelter et al. 2000; Anna and McKinley 2001; Nelson et al. 2011; Arnolds et al. 2010; Campo-Deaño and Clasen 2010; Vadillo et al. 2012; Keshavarz et al. 2015; Dinic et al. 2015).

Since the early 1990s, several devices have been developed to generate a uniaxial elongational deformation in viscoelastic fluids, and to quantify their extensional rheology (Galindo-Rosales et al. 2013). Considerable attention has been devoted to the filament stretching extensional rheometer (FiSER) (Anna and McKinley 2001; McKinley et al. 2001), and the capillary breakup extensional rheometer (CaBER^TM^) (McKinley and Tripathi 2000; Anna and McKinley 2001; Rodd et al. 2005). The FiSER device, developed after the seminal work of Matta and Tytus (1990), imposes an exponentially increasing separation of the end-plates, generating an extensional flow of constant deformation rate. A Versatile Accurate Deformation Extensional Rheometer (VADER-1000) was developed by Huang et al. (2016) for highly viscous samples (${\eta _{0} \gtrsim 10^{3}}$ Pa s) and is available as a new commercial extensional rheometer.

In the CaBER^TM^ apparatus, based on the pioneering work of Bazilevsky et al. (1981, 1990), the fluid is introduced between two cylindrical plates (typically between 4 and 6 mm in diameter) separated by a small distance, usually smaller than the plate diameter, to form a liquid bridge. Then, a rapid displacement of the upper plate is imposed, of the order of the plate diameter, which makes the liquid bridge unstable. The liquid bridge thins under the balance of capillary, viscous, elastic, inertial and gravitational forces. The velocity field far away from the end-plates (rods) is essentially one-dimensional and purely extensional (Schultz and Davis 1982).

As discussed by Galindo-Rosales et al. (2013), most of the extensional rheometers are suitable for operating only with high-viscosity fluids. Three notable exceptions developed recently are the Rayleigh-Ohnesorge Jet Elongational Rheometer (ROJER) (Sharma et al. 2015; Keshavarz et al. 2015), the optically-detected elastocapillary self-thinning dripping-onto-substract (ODES-DOS) extensional rheometer developed by Dinic et al. (2015), and the capillary thinning device developed by Vadillo et al. (2012), which was used to measure relaxation times as low as 80 μs of polystyrene in diethyl phthalate solutions. The ROJER device was used to investigate both the liquid extensional response and the effects of viscoelasticity on the atomization of dilute polyethylene oxide (PEO) solutions. In this extensional rheometer, the viscoelastic liquid jet is perturbed by a piezo-actuator at a prescribed frequency, while a stroboscopic imaging setup captures the motion of the liquid in slow motion, which allows a detailed analysis of the time evolution of the jet diameter during the breakup process. The ODES-DOS device was used to measure the response of aqueous dilute PEO solutions with small relaxation times (*λ*≲1 ms), and low viscosities (*η*_0_≲20 mPa s).

It is also important to highlight the works of Christanti and Walker (2001a, b, 2006), which used a method based on the work of Schümmer and Tebel (1982), to measure elongational properties of low viscosity fluids. The fluids were sprayed using an air atomizer, and the corresponding drop size distributions measured using a diffraction-based size analyzer. The results showed that viscoelasticity increases the mean drop diameter and a correlation between the measured relaxation times and the average droplet diameters was found.

Despite the variety of extensional rheometers developed so far, the HAAKE^TM^ CaBER^TM^ 1 (Thermo Scientific) is the leading edge apparatus commercially available which allows measuring the relaxation time and the extensional viscosity of dilute polymer solutions. According to Rodd et al. (2005), the minimum relaxation time measurable with this device is of the order of 1 ms. However, relaxation times of such a small magnitude are very difficult to measure, especially for low viscosity liquids, due to inertial effects and the short time for the filament thinning and breakup. Campo-Deaño and Clasen (2010) developed the so-called slow retraction method (SRM) by combining the CaBER^TM^ 1 apparatus with high-speed imaging. In this technique, the filament thinning is promoted by a slow extension of the liquid bridge, contrary to the fast step strain of the conventional technique. Using the slow retraction method, inertial effects were minimized, and relaxation times as low as 240 μs were measured for aqueous solutions of PEO (Campo-Deaño and Clasen 2010). Solvent evaporation in volatile fluids or water absorption in hygroscopic liquids may play a significant role in the measurements using this method, and these effects can change the polymer concentration during the quasi-static liquid bridge stretching, depending on the solution volatility or hygroscopicity.

In this work, we describe a miniaturized filament breakup device combining the slow retraction method and high-speed imaging techniques. A slow linearly increasing separation of the end-plates is used to trigger the elongational flow. In order to reduce solvent loss by evaporation or water absorption of hygroscopic solutions, the filament thinning and breakup takes place in an immiscible oil, which can also be useful to perform particle tracking to measure the velocity field in the thinning filament. We measure the extensional relaxation time of aqueous polyacrylamide solutions (PAA) over a wide range of concentrations, including ultra-dilute polymer solutions. Additionally, two PEO solutions matching those used recently by Keshavarz et al. (2015) in the ROJER device were used in the present investigation in order to validate the experimental technique and show its applicability for measuring relaxation times below 1 ms.

## Experimental method

### Experimental setup

**Fig. 1:**
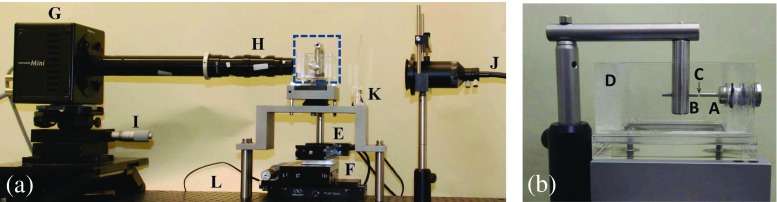
**a** Photograph of the experimental setup. **b** Photograph of the experimental cell (zoomed view of the dashed blue rectangle in (**a**)). Static rod (*A*), mobile rod (*B*), liquid bridge (*C*), transparent tank (*D*), motorized traverse (*E*), translation stage (*F*), high-speed camera (*G*), optical lenses (*H*), triaxial translation stage (*I*), optical fiber (*J*), frosted diffuser (*K*), and optical table (*L*)

Figure 1 shows an overall view of the experimental setup used in this work, and a zoomed view of the device where the fluid is tested. A horizontal liquid bridge is formed between two cylindrical rods. The diameter of the rods used in the experiments was *d*_0_ = 2.0 mm. We also tested rods with *d*_0_ = 1.0 mm, but if necessary even smaller diameters can be used, reducing inertial and gravitational effects and the required sample volume. One of the rods (A) is fixed, while the other (B) can be displaced along its axial direction. A volume of liquid (C) is introduced in the gap between the two rods using a feeding flexible capillary (400 μm in diameter) connected to a syringe. In the experiments with an outer liquid bath, both the rods and the liquid bridge are submerged in a transparent perspex tank (D), filled with another immiscible liquid. The mobile rod is connected to a motorized stage (Zaber Tech T-LSM100A) (E) to control its distance from the static rod. The motorized traverse was mounted on a manual high-precision translation stage (F) to ensure the correct alignment of the two rods.

Digital images of the liquid bridge with a resolution of 1280 × 1000 pixels were acquired with a high-speed CMOS camera (Fastcam mini UX100) (G), operated typically at 5000 frames per second (fps), using an exposure time of 50 μs. To measure relaxation times below 1 ms, the camera frame rate was increased up to 40,000 fps, while the spatial resolution and the exposure time were decreased down to 1280 × 120 pixels and 5 μs, respectively. The camera was connected to a set of optical lenses (H) (Optem Zoom 70 XL) with variable magnification from 1 × to 5.5 ×. The resulting magnification varied between 3.44 and 0.624 μm/pixel. The camera could be displaced both horizontally and vertically using a triaxial translation stage (I) to focus the liquid bridge in the field of view. The fluid was illuminated from the back side with white light provided by an optical fiber (J) connected to a metal halide light source (Leica EL6000). The optical fiber was connected to a set of focusing lenses, providing a focused light beam approximately 25 mm in diameter. A frosted diffuser (K) was placed between the optical fiber and the cell to provide a uniform illumination. All these elements were mounted on the top of an optical table (L) to reduce vibrations. A micro-thermocouple (100 μm in diameter) was used to measure the temperature of the PEO and PAA liquids, which was *T* = 20 ± 1 °C and 25 ± 1 °C, respectively.

### Experimental protocol

**Fig. 2:**
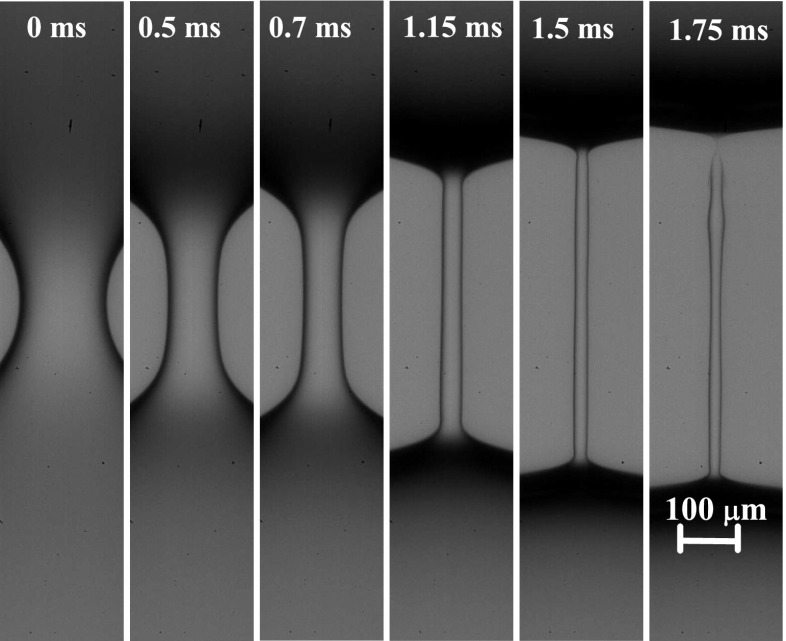
Temporal evolution of a liquid filament of polyacrylamide in water with a concentration of 5 ppm and immersed in the 0.65 cSt silicone oil

The extensional relaxation times of the polymeric solutions were measured using the following procedure. A liquid droplet was gently placed between the two rods located in the empty tank, creating a liquid bridge about 500 ± 50 μm in length with the triple contact lines anchored to the edges of the supporting rods. In the experiments with an outer liquid bath, the tank was subsequently filled with an immiscible oil until the liquid bridge was completely submerged in the bath. To induce the filament thinning, we used the slow retraction method. For this purpose, the mobile rod was displaced at a constant velocity of 5 μm/s while the other rod remained fixed. This speed was low enough for the liquid bridge to undergo a sequence of equilibrium states, until the instability occurs spontaneously above a critical distance between the rods (Slobozhanin and Perales 1993; Campo-Deaño and Clasen 2010). The instant *t* = 0, illustrated in Fig. 2, corresponds approximately to the onset of the instability. The subsequent filament thinning and breakup was recorded using the high-speed camera, as illustrated in Fig. 2 for some representative times.

The images acquired in the course of the experiments were processed to detect the filament interface with a sub-pixel resolution technique (Ferrera et al. 2006; Vega et al. 2009). The diameter of the filament at its mid-plane between the upper and lower filament ends, *d*_*mid*_, and the minimum diameter along the filament axis, *d*_min_, were determined for each image. Representing the diameter data as function of time in a semi-log plot, it is possible to identify the time interval within which the diameter decays exponentially for viscoelastic fluids. This interval corresponds to the elasto-capillary regime, where the balance between surface tension and tensile stresses produces the homogeneous stretching of a quasi-cylindrical thread. The time evolution of the filament diameter in this regime was fitted by the exponential function (Bazilevsky et al. 1990)

1 $$\vspace*{-1pt} d_{\min}(t)= A\ \exp{[-t/(3\lambda)]}, $$

which allows the calculation of the extensional relaxation time, *λ*. Each experiment was performed five times to assess the reproducibility and to estimate the standard deviation of the measured relaxation times.

The pathlines and velocity of some tracer particles suspended in the liquid filament were measured using particle tracking velocimetry (PTV). For this purpose, polystyrene tracer particles with density *ρ* = 1055 kg/m^3^ and with 2 μm average diameter were introduced in the liquid before the experiment. The presence of an outer bath with a refractive index close to that of the working fluid minimizes refraction of light in the cylindrical fluid filament, allowing to detect precisely the position of the tracer particles in the acquired images. The particles in the fluid filament essentially moved in the axial direction, as expected for a purely extensional flow (Gier and Wagner 2012). The optical distortion produced by the quasi-cylindrical interface in the axial direction is negligible. The tracking of the position of the particles over time was performed with the opensource 3D computer graphics software Blender (version 2.73a), using its feature tracking capabilities and a Python script to determine the coordinates of the particles along time.

### Fluids

The test fluids used in the experiments were polymeric solutions of PEO (Sigma-Aldrich, *M*_*w*_ = 10^6^ g/mol) in a mixture of glycerol/water (40/60 wt. %) and of polyacrylamide (Polysciences, *M*_*w*_ = 18 × 10^6^ g/mol) in water. Stock solutions with concentration *c* were prepared by dissolving the polymers in the solvent by agitation with a magnetic stirrer at low angular speeds, to minimize mechanical degradation of the long polymer chains. The PEO concentrations of the two solutions used were 100 ppm (PEO100) and 500 ppm (PEO500), which match those used by Keshavarz et al. (2015) in the ROJER device. The PAA concentrations ranged between 2 and 1000 ppm. Experiments were conducted both in air and using a liquid bath to minimize solvent evaporation. Silicone oils (SO) with kinematic viscosities ranging from 0.65 to 35 cSt were used to form the liquid bath.

**Fig. 3:**
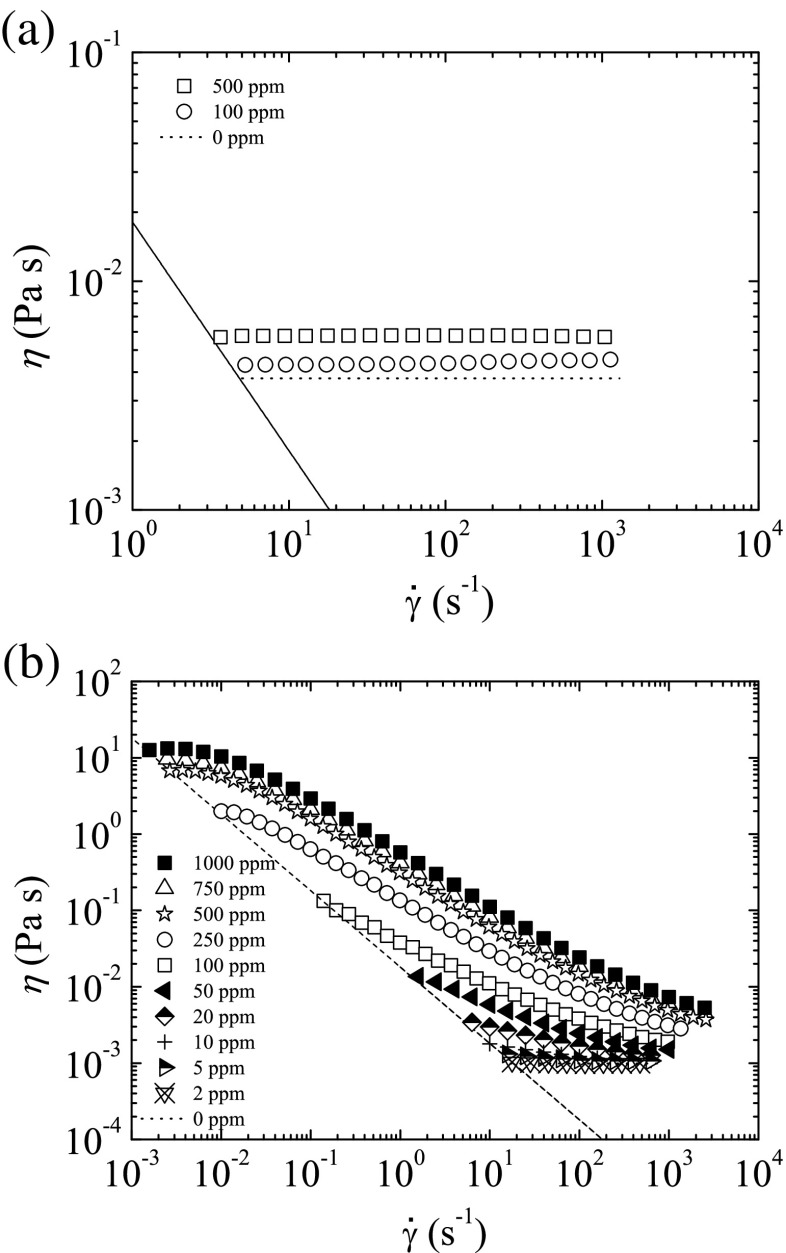
Variation of the shear viscosity *η* with shear rate $\dot {\gamma }$ for **a** PEO (*T* = 20 °C) and **b** PAA (*T* = 25 °C) solutions. The concentrations of PEO in a glycerol/water mixture (40/60 wt. %) were 100 and 500 ppm. The concentrations of PAA in water varied between 2 and 1000 ppm. The *dotted horizontal lines* represent the shear viscosity of the solvents. The *dashed lines* indicate the minimum measurable shear viscosity based on 20 × the minimum resolvable torque of the shear rheometer used

The variation of the shear viscosity, *η*, with shear rate, $\dot {\gamma }$, was measured for all PEO and PAA polymer solutions using a shear rheometer (Physica MCR301, Anton Paar) with a cone-plate geometry with 75 mm diameter and 1° angle. As shown in Fig. 3, shear-thinning in the PAA solutions becomes more pronounced when the polymer concentration increases. This effect is more noticeable for *c*>50 ppm, close to the overlap concentration (Sousa et al. 2015). On the other hand, the shear viscosity of the PEO solutions is nearly independent of the shear rate due to the low concentrations used in the experiments, well below the overlap concentration, *c* ^∗^ = 1400 ppm (Keshavarz et al. 2015). The surface tension *σ* was measured with the TIFA method (Cabezas et al. 2005), and is shown in Table 1 for different oil/water systems. The refractive index *n* was measured using an Abbe refractometer (WYA-2S, Optic Ivymen System). The corresponding values for the different oils are listed in Table 1, while the values for the PAA solutions and distilled water are shown in Table 2.

**Table 1:** Surface tension, *σ*, for water-oil interfaces and refractive index, *n*, of the oils at *T* = 25 °C

Liquid	0.65 cSt SO	1 cSt SO	2.78 cSt SO	5 cSt SO	35 cSt SO
*σ* (mN/m)	43.2	42.9	41.0	39.0	37.7
*n*	1.375	1.384	1.385	1.399	1.403

**Table 2:** Refractive index, *n*, of the PAA solutions and distilled water measured at *T* = 25 °C

Fluid	*n*	Fluid	*n*
PAA1000	1.3331	PAA20	1.3324
PAA750	1.3325	PAA10	1.3324
PAA500	1.3326	PAA5	1.3324
PAA250	1.3328	PAA2	1.3324
PAA100	1.3325	Distilled water	1.3325
PAA50	1.3324		

## Results and discussion

**Fig. 4:**
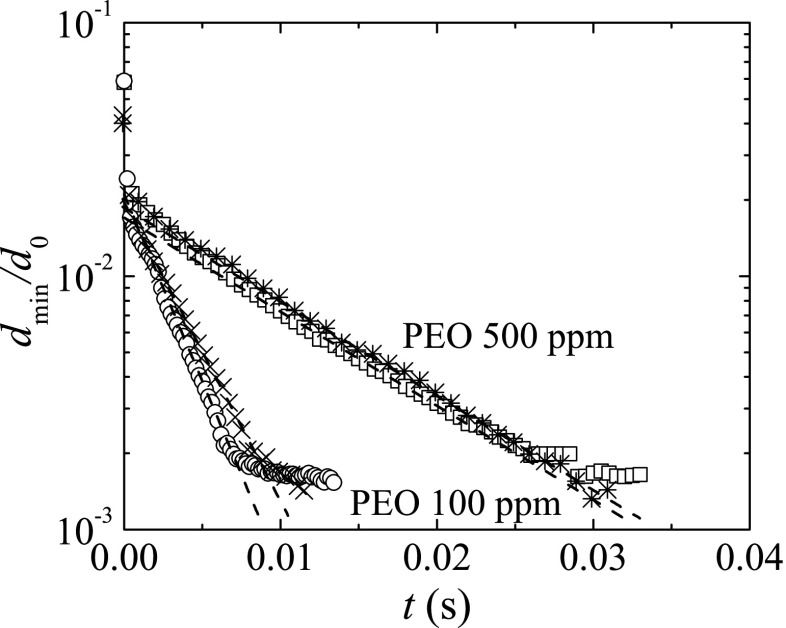
Filament diameter $d_{\min }/d_{0}$ as a function of time for the PEO solutions surrounded by air ($\bigcirc , \square $) and the 0.65 cSt SO (×, ∗) measured at *T* = 20 ± 1 °C. The extensional relaxation times measured in air are *λ* = 1.01 ± 0.03 ms and 3.9 ± 0.3 ms for PEO100 and PEO500, respectively, while the extensional relaxation times measured in the silicone oil are *λ* = 1.27 ± 0.04 ms and 3.89 ± 0.01 ms for PEO100 and PEO500, respectively. For clarity, in the case of PEO100, we only plot one quarter of the points, while for the PEO500 we only plot one tenth of the points. The *dashed lines* indicate the fits to obtain the relaxation times. The diameter of the rods is *d*_0_ = 2 mm

Figure 4 shows the time evolution of the ratio between the minimum filament diameter, *d*_min_, and the rod diameter, *d*_0_, for the PEO solutions surrounded by air and also in an oil bath of the less viscous silicone oil. We consider the minimum diameter of the filament instead of the filament diameter at the middle plane between the upper and lower filament ends. The elasto-capillary regime occurs for about one decade of decrease of *d*_min_/*d*_0_. The extensional relaxation times measured in air for PEO100 and PEO500 fluids at *T* = 20 ± 1 °C were *λ* = 1.01 ± 0.03 ms and 3.9 ± 0.3 ms, respectively, which are in good agreement with the results obtained by Keshavarz et al. (2015) in their recent investigation using the ROJER device. The small differences between both measurements (1 % and 28 % for PEO100 and PEO500, respectively) are probably due to the variability in the polymer batches and small temperature differences in the two works. The same measurements were done again at *T* = 20 ± 1 °C using the less viscous oil as outer fluid, and the results presented in Fig. 4 show a similar behavior and the corresponding extensional relaxation times measured were *λ* = 1.27 ± 0.04 ms and 3.89 ± 0.01 ms for PEO100 and PEO500 fluids, respectively. The slow retraction method implemented in this work in a miniaturized device, together with the use of a low viscosity oil bath provides reliable measurements of the relaxation time even below 1 ms, as will be shown.

Beads-on-a-string structures were formed over a substantial time interval of the final stage of the filament thinning process for the PEO solutions tested. This phenomenon is indirectly illustrated in Fig. 4, since the dimensionless diameter becomes nearly constant for $t \gtrsim $ 10 ms and 30 ms for PEO100 and PEO500, respectively. The competition between capillary, elastic, and inertial forces leads to the formation of an array of beads connected axially by thin filaments. The onset of this type of instability is commonly observed for dilute solutions of high molecular weight flexible polymers. In particular, PEO solutions, which have been extensively used in the investigation of the capillary thinning and breakup of viscoelastic samples, frequently form these structures when stretched (Oliveira and McKinley 2005; Rodd et al. 2005; Tirtaatmadja et al. 2006).

**Fig. 5:**
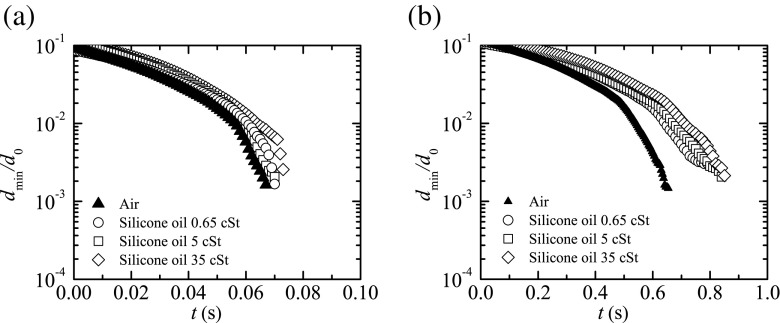
Minimum filament diameter $d_{\min }/d_{0}$ as a function of time for fluids PAA100 (**a**) and PAA750 (**b**) surrounded by air (*solid symbols*) and silicone oils with different viscosities (*hollow symbols*). For clarity, in all cases, we only plot one point per each five images. The diameter of the rods used is *d*_0_ = 2 mm

The applicability of a capillary breakup extensional rheometer to low-viscosity fluids can be improved by using the slow retraction method and making use of smaller plate diameters, because inertial and gravitational effects are minimized in this way. However, during the slow retraction of the moving plate, the evaporation of volatile solvents or absorption of water vapor by hygroscopic fluids may produce misleading results. To reduce such an undesirable effect, the liquid bridge of the PAA polymer solutions tested was submerged in an oil bath. Figure 5 shows the temporal evolution of the filament diameter for fluids PAA100 and PAA750 submerged in 0.65 cSt, 5 cSt, and 35 cSt silicone oils. As can be observed, the elasto-capillary regime was reached for $t\gtrsim $ 0.03 and 0.3 s for fluids PAA100 and PAA750, respectively. The extensional relaxation times determined are nearly independent of the outer bath viscosity, despite the wide range of viscosities of the oils used in the tests, covering a variation of nearly two orders of magnitude. In the experiments with air, the liquid bridge evolution was slightly faster, and the exponential thinning of the filament occurred earlier, but the slopes of the linear regions where the elasto-capillary regime was reached are comparable, and consequently the corresponding relaxation times are also similar. To allow a better comparison of the results, the curves were shifted in time to obtain the elasto-capillary regime in similar time ranges for each polymer concentration. To minimize water evaporation in the experiments in air, particularly during the initial slow stretch of the filament, it is recommended to add in advance some water in the bottom of the reservoir (transparent tank D in Fig. 1) to saturate the environment in the vicinity of the filament.

**Fig. 6:**
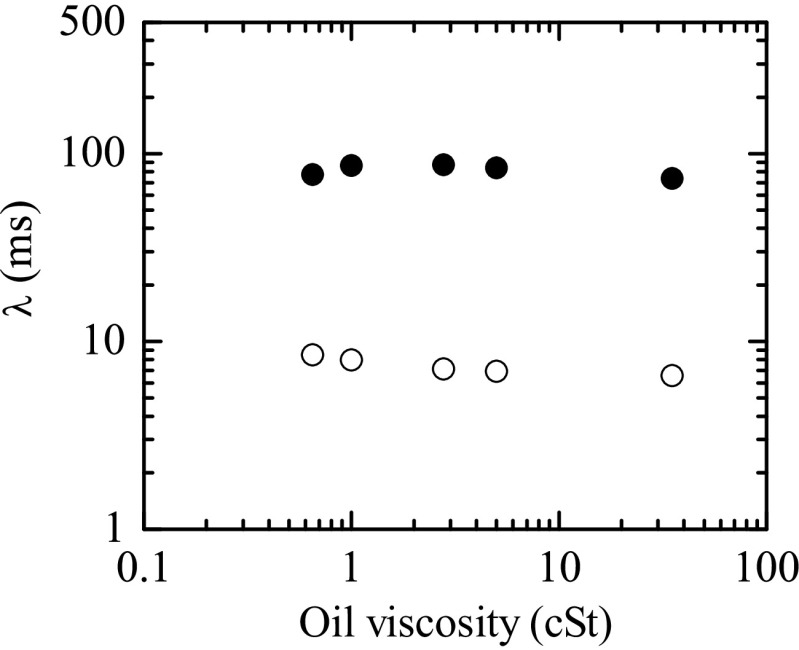
Extensional relaxation time *λ* as a function of the oil bath kinematic viscosity for two PAA aqueous solutions: *c* = 750 ppm (*solid symbols*); *c* = 100 ppm (*hollow symbols*)

In order to assess the influence of the outer bath viscosity on the filament thinning of the PAA solutions, experiments for *c* = 100 and 750 ppm were done with five different oil baths with kinematic viscosities varying between 0.65 and 35 cSt. In all cases, the elasto-capillary regime was observed over a significant time interval, and the corresponding extensional relaxation times were determined (Fig. 6). The outer bath viscosity did not affect significantly the extensional relaxation times measured in the range of oil viscosities tested. Nevertheless, in the following experiments using an outer oil bath, we will always use the less viscous silicone oil, to minimize the shear stress in the interface.

**Fig. 7:**
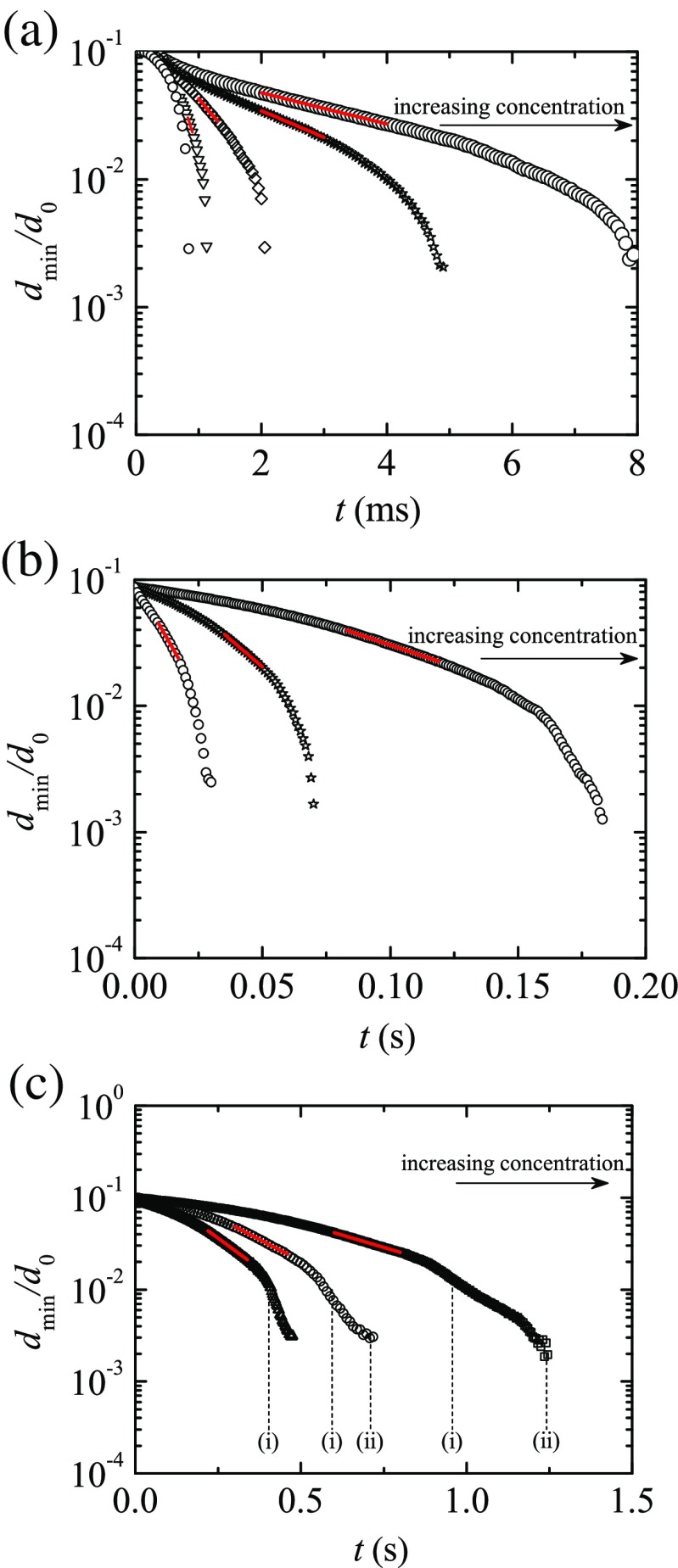
Minimum filament diameter *d*_*min*_/*d*_0_ as a function of time for different concentrations of PAA in water surrounded by 0.65 cSt silicone oil. The PAA concentrations are **a** *c* = 0, 2, 5, 10, 20 ppm; **b** *c* = 50, 100, 250 ppm; **c** *c* = 500, 750, 1000 ppm. The PAA concentration increases in the direction indicated by the *arrows*. For the higher concentrations, the onset of a single and multiple beads during the filament thinning is indicated by labels (*i*) and (*ii*), respectively. The *red lines* indicate the fits to determine the extensional relaxation times. For clarity, we only plot one fifth of the experimental points in (**b**) and (**c**). The curves were shifted in time to match at short times. The diameter of the rods used is *d*_0_ = 2 mm

**Fig. 8:**
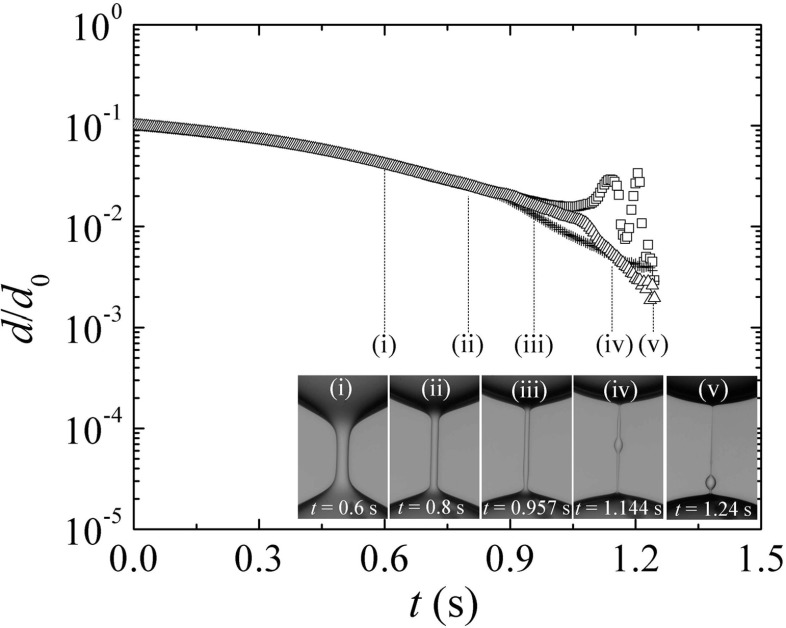
Filament diameter at the mid-plane between the upper and lower filament ends (*squares*), and the minimum diameters of the upper (*triangles*) and lower (*crosses*) filament ends for PAA1000 fluid in the 0.65 cSt silicone oil. The images (*i*) and (*ii*) correspond to the elasto-capillary regime, (*iii*) and (*iv*) to the formation of a single bead, and (*v*) to the onset of multiple beads. The diameter of the rods used is *d*_0_ = 2 mm

Figure 7 shows the time evolution of the filament diameter for the PAA aqueous solutions surrounded by the 0.65 cSt silicone oil at *T* = 25 °C. The concentration of PAA, and consequently the relaxation time, increases in the arrow direction. In all the cases (except for the solvent), an elasto-capillary regime was identified (indicated in the figure by the red lines), and the relaxation time was determined by fitting (1) to the variation of the filament minimum diameter as function of time. For the solutions with higher concentrations, the filament becomes asymmetric relative to the mid-plane between the upper and lower filament ends due to the formation of a bead near the centre of the filament, followed by the onset of multiple beads along the thread. These events occurred at the times indicated in Fig. 7c, and are illustrated in Fig. 8.

Figure 8 shows simultaneously the filament diameter at the mid-plane and the minimum diameters of the upper and lower filament ends for PAA1000 fluid in the 0.65 cSt silicone oil. The extensional relaxation time was measured when the filament exhibited a quasi-cylindrical shape and the three diameters almost coincided [insets (i) and (ii)]. At *t* ≃ 0.9 s, the shape of the filament changes and is no longer cylindrical [inset (iii)]. Then, a single bead appears [inset (iv)] followed by multiple small beads [inset (v)]. After the onset of these instabilities, the filament diameter is no longer uniform, and the results are not reliable for the measurement of *λ*. Therefore, to determine the extensional relaxation time, we restricted the analysis of the filament only when it presented a quasi-cylindrical shape.

**Fig. 9:**
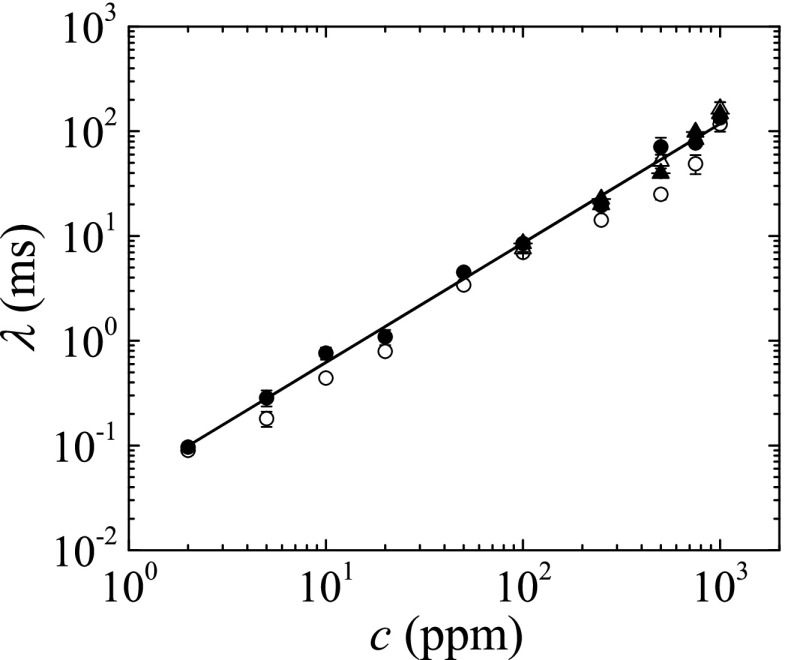
Extensional relaxation time *λ* as a function of the polymer concentration *c* for aqueous solutions of PAA at *T* = 25 °C. The *solid and hollow circles* are the results obtained using the device developed in this work with liquid bridges surrounded by 0.65 cSt silicone oil and air, respectively. The *solid and hollow triangles* are the results measured with the CaBER^TM^ 1 apparatus using the slow retraction method and the standard rapid stretching process, respectively. The *line* represents the fit to the data: *λ* [ms] =0.045(*c* [ppm])^1.14^

Figure 9 shows a comparison between the extensional relaxation times measured using four techniques: the slow retraction method implemented in our device with and without an outer immiscible liquid bath, the slow retraction method implemented in the commercial CaBER^TM^ 1 device (with a plate separation speed of 0.18 mm/s), and the standard fast stretching procedure also implemented in the commercial rheometer CaBER^TM^ 1. In this last case, the rods were separated exponentially for 50 ms from 2 mm to a final distance between 5.63 and 7.42 mm, depending on the fluid tested. The rods in the CaBER^TM^ 1 apparatus were 4 mm in diameter. Inertial effects prevented us from obtaining reliable results with the CaBER^TM^ 1 device for relaxation times below about 10 ms, which corresponds to PAA concentrations below 100 ppm, approximately. Inertial effects were also relevant when the slow retraction method was implemented in the CaBER^TM^ 1 device owing to the large diameter of the rods. Overall, there is good agreement between the results obtained with the CaBER^TM^ 1 device and the measurements with the device developed in this work, particularly for the cases that use an outer liquid bath.

The results obtained with the PAA aqueous solutions exhibit a power law dependence upon the concentration (*λ* [ms] = 0.045(*c* [ppm])^1.14^), with a power law exponent close to one, in agreement with previous works (Clasen et al. 2006; Arnolds et al. 2010).

**Fig. 10:**
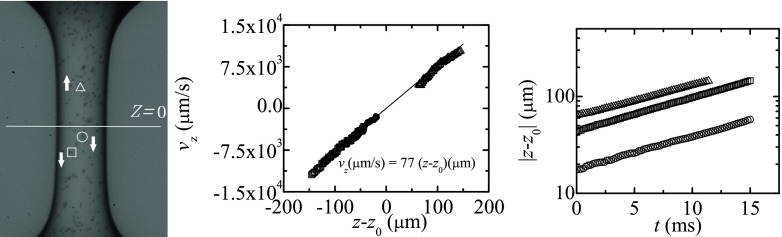
Axial velocity component *v*_*z*_ (*middle graph*) and distance |*z* − *z*_0_| from the stagnation point (*right graph*). The experiment was conducted with a solution of 100 ppm of PAA in water surrounded by 0.65 cSt silicone oil. The symbols in the graphs correspond to those labeling the tracked particles in the left image

**Fig. 11:**
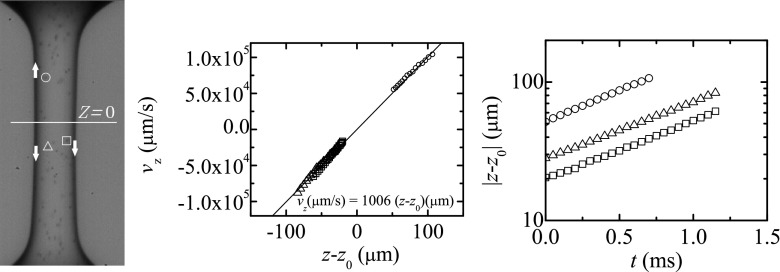
Axial velocity component *v*_*z*_ (*middle graph*) and distance |*z* − *z*_0_| from the stagnation point (*right graph*). The experiment was conducted with a solution of 10 ppm of PAA in water surrounded by 0.65 cSt silicone oil. The symbols in the graphs correspond to those labeling the tracked particles in the left image

Particle tracking velocimetry (PTV) was used to track the position of several particles as function of time and computing their velocity in the liquid filament during the elasto-capillary regime. Figures 10 and 11 show the axial velocity component, *v*_*z*_, and the axial distance, *z* − *z*_0_, from the stagnation point position, *z*_0_, of three of the particles tracked in the course of the experiments. These particles were located next to the filament symmetry axis to minimize optical distortion, although we emphasize that the use of the oil bath minimizes light refraction, and also the streamwise location of the particle is not affected by the radial curvature of the liquid filament. It must be noted that the stagnation point does not necessarily lie on the filament midplane (Gier and Wagner 2012). We consistently calculated its position *z*_0_ as the location where the linear fit to the data {(*z* − *z*_0_, *v*_*z*_)} has zero velocity at *z* = *z*_0_. Our results show that *v*_*z*_ is approximately given by the uniform uniaxial extensional flow, $v_{z}=\dot {\varepsilon }(z-z_{0})$, where $\dot {\varepsilon }$ is the constant elongation rate in the elasto-capillary regime. As expected, the particle position, |*z*(*t*)−*z*_0_|, represented in a semi-log plot is close to a straight line, and the elongation rate can be computed from the slope of such line. The average values obtained from Figs. 10 and 11 are $\dot {\varepsilon }= 77$ s^−1^ and 1006 s^−1^ for *c* = 100 and 10 ppm, respectively. These values are consistent with those obtained from the relaxation times measured from the filament diameter decay as function of time: $\dot {\varepsilon }=2/(3\lambda ) \approx 80$ s^−1^ and 900 s^−1^ for *c* = 100 and 10 ppm, respectively.

The fact the inner and outer liquids can have similar refraction indices is a significant advantage of the use of an immiscible oil bath in our setup, allowing to easily track the position of tracer particles in the fluid filament during the extensional thinning. That feature can also be exploited to visualize the deformation, due to extensional flow, of flexible components of the tested fluid, such as red blood cell deformation in blood or DNA stretching. In these cases, the visualization of the fluid elements is better achieved using fluorescence imaging by means of laser illumination and an appropriate barrier filter or dichroic mirror to block the laser light between the fluid filament and the high-speed camera sensor, similar to the setup used by Gier and Wagner (2012). In this way, only the fluorescence emitted by adequate fluochromes attached to the fluid components is allowed to reach the camera sensor.

## Conclusions

A setup for monitoring the filament thinning and breakup of a liquid placed between two cylindrical rods was developed, combining the slow retraction method with high-speed imaging techniques. The use of an immiscible oil bath allows to reduce solvent loss by evaporation of volatile liquids, or water absorption in hygroscopic fluids. Gravitational and inertial effects are minimized, due to the use of small diameter cylindrical rods and a slow retraction of the moving rod to induce the filament thinning, allowing reliable measurements of the extensional relaxation time of dilute polymer solutions down to about 100 μs. Using a low viscosity immiscible oil bath with a refractive index similar to the test fluid allows to use particle tracking velocimetry to measure the velocity of tracer particles and the corresponding elongation rate of the thinning filament, confirming the extensional relaxation times measured from the exponential decay of the filament diameter.
